# Recurrent High-Grade Retroperitoneal Sarcoma: A Case Report

**DOI:** 10.7759/cureus.79965

**Published:** 2025-03-03

**Authors:** Peter Richa, James R Conomea, Cade Mullins, M. Rudwan Soukieh, Ravish Narvel

**Affiliations:** 1 School of Medicine, Lake Erie College of Osteopathic Medicine, Bradenton, USA; 2 Internal Medicine, Ascension St. Vincent's Riverside, Jacksonville, USA

**Keywords:** high-grade sarcoma, liposarcoma, recurrent sarcoma, retroperitoneal, retroperitoneal sarcoma surgery, sarcoma recurrence, sarcoma soft tissue, undifferentiated sarcoma

## Abstract

Retroperitoneal sarcomas are rare and challenging soft tissue tumors that can have an insidious presentation. These tumors may present asymptomatically or display vague symptoms such as nausea, abdominal pain, and constipation. Rapid detection and treatment of these tumors are necessary as they may metastasize or compress adjacent structures within the body. Imaging and biopsy play a critical role in diagnosing and managing retroperitoneal sarcomas, with treatment decisions largely influenced by the tumor's histological subtype. Due to the high recurrence rate of these tumors, adjuvant therapies and consistent monitoring are essential. This case report discusses the recurrence of a high-grade liposarcoma in a 73-year-old male patient status post retroperitoneal sarcoma resection. Despite initial successful surgical intervention, the patient's non-compliance with follow-up care led to a recurrence, highlighting the importance of long-term monitoring, adherence to post-surgical protocols, and multidisciplinary decision-making.

## Introduction

Sarcomas are defined as soft tissue malignancies derived from mesenchymal tissues [1]. They are named for the tissue they arise from, with liposarcomas (LPS) arising from adipose connective tissue cells. LPS is further differentiated into subtypes by its degree of differentiation: well-differentiated, dedifferentiated, myxoid, pleomorphic, and mixed [2]. Soft tissue sarcomas make up <1% of malignancies in adults [3]. LPS account for 20% of all soft tissue sarcomas and commonly develop in the extremities [4]. Retroperitoneal LPS (RPLPS) is a rare manifestation of the disease and occurs in 13% of LPS cases [1]. The five-year survival rate of a well-differentiated LPS (WDLPS) is above 90%, while the five-year survival rate of a dedifferentiated LPS (DLPS) is below 75% [2]. The prognosis is dependent on tumor grading and characteristics [5].

RPLPS classically present asymptomatically until they grow to a size that compresses surrounding organs and structures [2]. If a patient does endorse symptoms, it is typically vague such as non-localized abdominal discomfort [2]. The retroperitoneal space is quite large, allowing RPLPS to remain undetected for prolonged periods. Due to their asymptomatic nature, 50% of RPLPS exceed 20 cm in size at diagnosis [5]. A biopsy is regarded as the gold standard for diagnosing RPLPS [2]. However, imaging is widely accepted as a means of diagnosis due to the risks of peritoneal implants during biopsy [2,5]. Computed tomography (CT) and magnetic resonance imaging (MRI) scans are commonly used during presurgical workups to determine tumor resectability [2]. 

The mainstay treatment of RPLPS is surgical resection. Resection of RPLPS can be uniquely difficult due to indistinct margins [3]. Typical surgical resections include seeded adjacent structures, while more aggressive approaches can include partial resection of uninvolved structures [2]. Due to the chance of recurrence, current treatment guidelines recommend post-resection adjuvant therapy, including radiotherapy, systemic chemotherapy, and targeted therapy [6]. Current National Comprehensive Cancer Network guidelines for retroperitoneal sarcoma (RPS) recommend that patients follow up with their oncologist for examination with imaging every 3-6 months for 2-3 years, then every six months for the following two years, and then annually [7]. The following case is a presentation of RPLPS recurrence after surgical resection, in which the patient was non-compliant with adjuvant follow-up. It is intended to serve as a review of the diagnostic and treatment modalities currently available for RPLPS.

## Case presentation

A 73-year-old Caucasian male patient presented to the emergency department with complaints of worsening abdominal pain. Past medical history and relevant comorbidities included tobacco use, hypertension, hyperlipidemia, paroxysmal atrial fibrillation with rapid ventricular response, heart failure with reduced ejection fraction, coronary artery disease, and RPS status post resection and colostomy two years ago. The patient noted associated fever and constipation for six days, with laxatives providing no relief.

**Figure 1:**
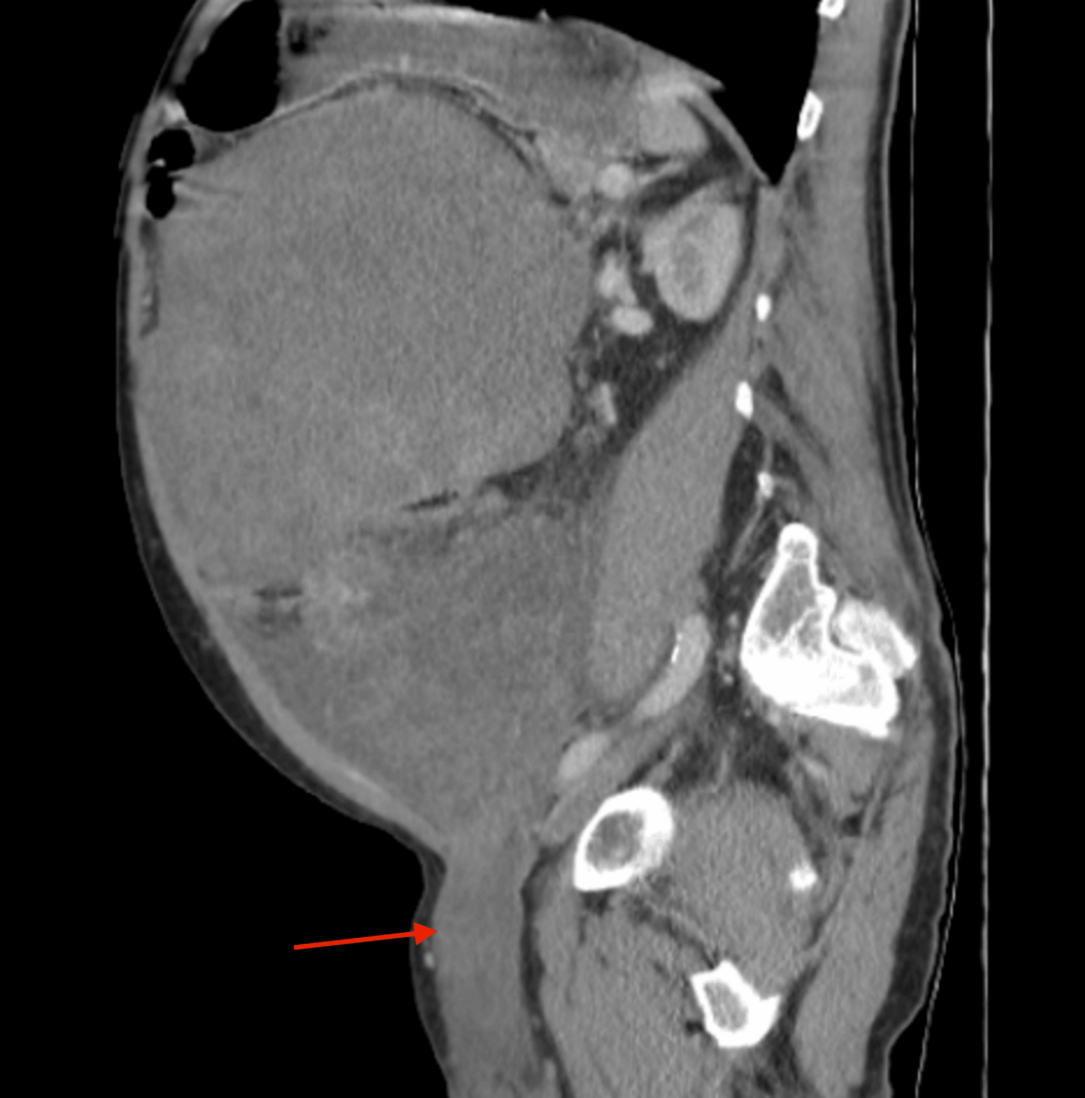
Sagittal CT view of the abdomen and pelvis, showing the large intra-abdominal pelvic mass extending into the left inguinal canal (red arrow) CT: computed tomography

Two years ago, the patient presented to the emergency department with complaints of fever, cough, dizziness, left lower quadrant pain, and testicular swelling. His lab values at the time revealed hyponatremia, anemia, and leukocytosis. Diagnostic imaging, including an abdominal CT scan and MRI scan, showed a large left abdominal mass extending into the left inguinal canal with trace-free fluid in the pelvis (Figure 1). An ultrasound-guided core needle biopsy was performed, and pathology characterized the mass as a grade 2 leiomyosarcoma. After consultations with urology, general surgery, and oncology, an en bloc resection of the 50×50 cm RPS, including the left testicle, sigmoid colon, and rectum, took place approximately two weeks later. The pathology report of the surgical specimen characterized the RPS as a grade 3 DLPS. Furthermore, the report specified that the colon resection and mesenteric margins are uninvolved, with multifocal involvement of the soft tissue resection margins. Approximately six months later, the patient underwent elective adhesiolysis and resection of the previously placed end colostomy. It was recommended that the patient follow up with oncology for adjuvant chemotherapy to minimize the chances of recurrence.

**Figure 2:**
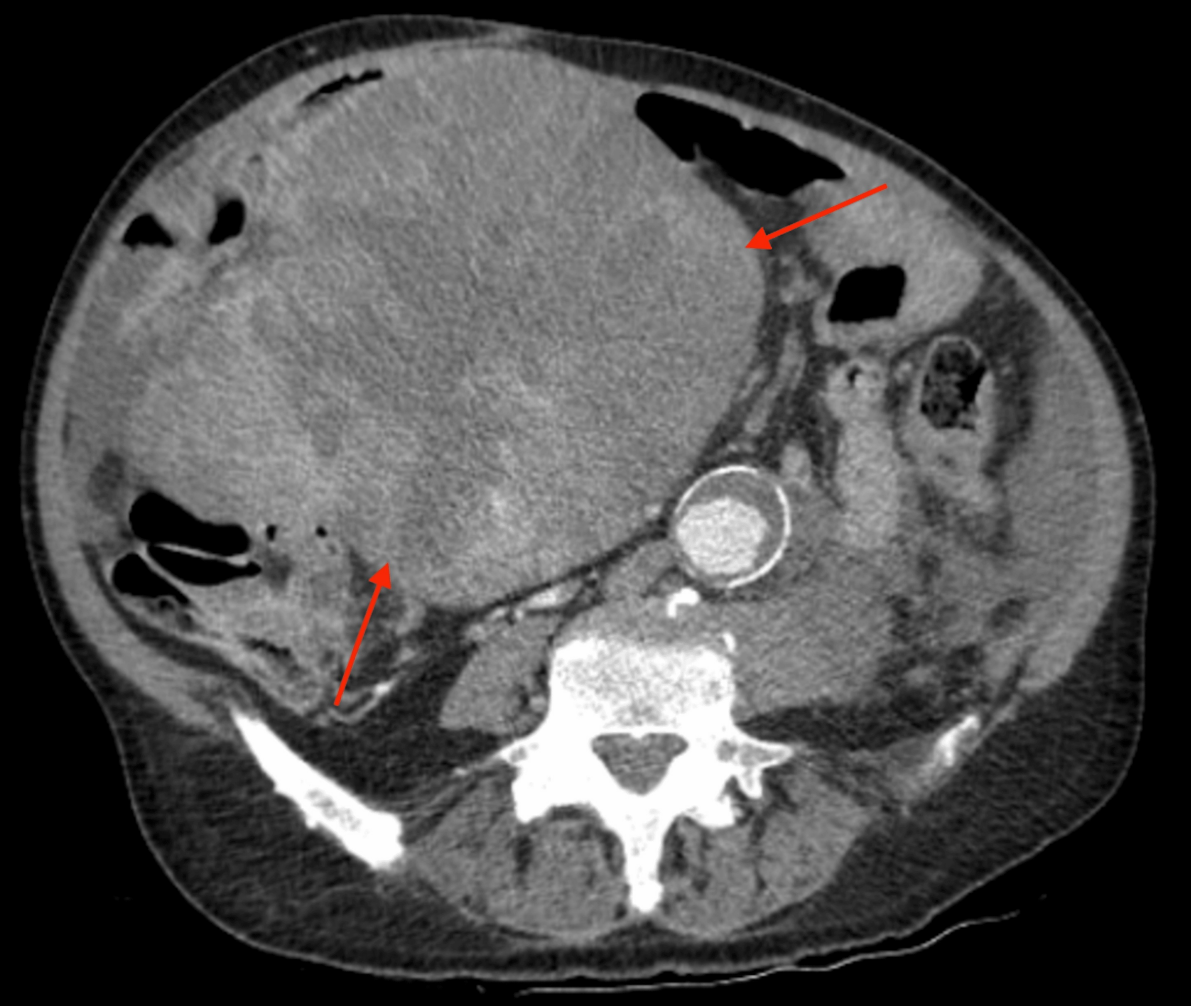
Axial CT view of the abdomen and pelvis, showing the large complex soft tissue mass (red arrows) extending from the right upper quadrant into the lower abdomen CT: computed tomography

**Figure 3:**
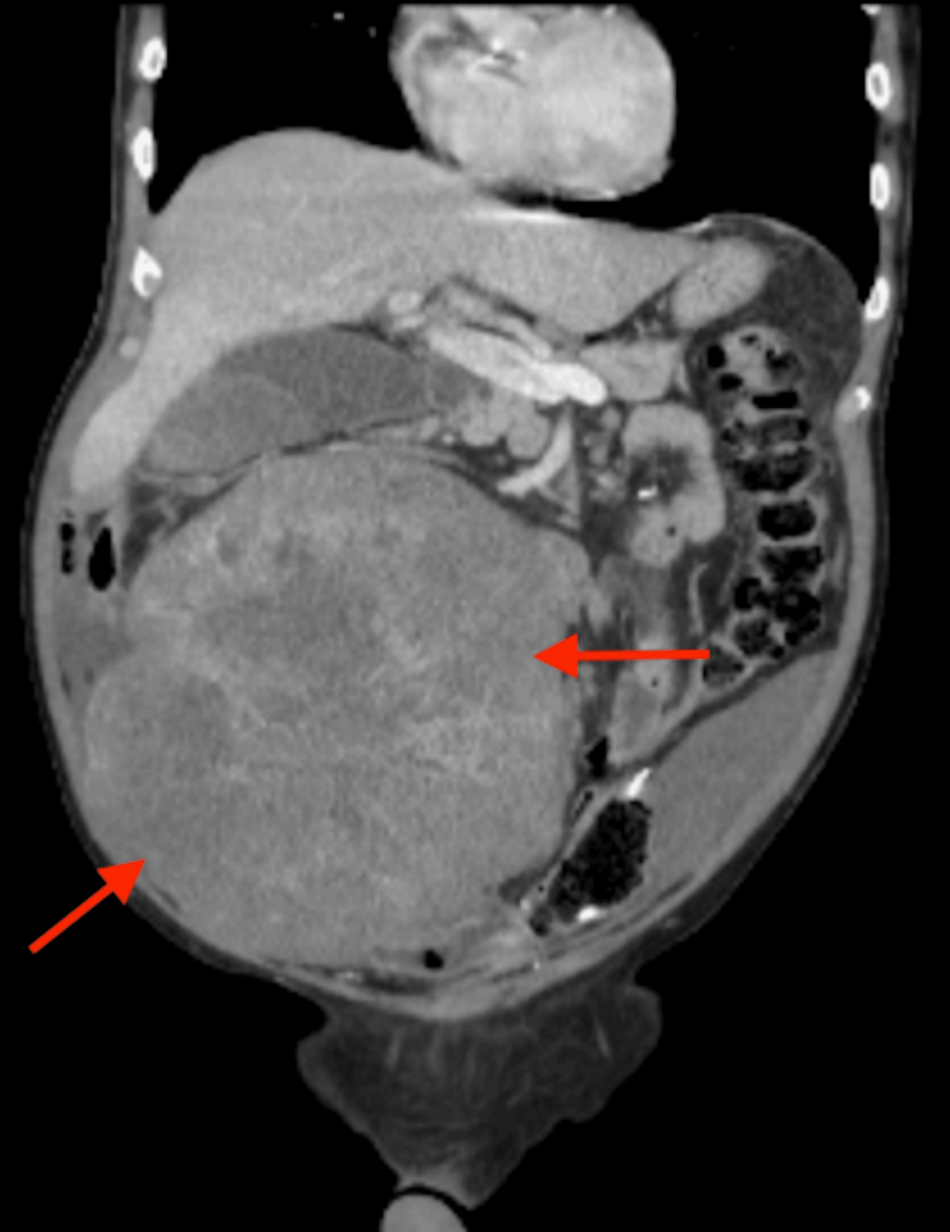
Coronal CT view of the abdomen and pelvis, showing the large complex soft tissue mass (red arrows) extending from the right upper quadrant into the lower abdomen CT: computed tomography

Two years later, the patient presented to the emergency department with symptoms previously described. Once again, the patient's lab values demonstrated hyponatremia, leukocytosis, and anemia. An abdominal CT scan showed a large central mesenteric mass extending from the right upper quadrant into the lower pelvis (Figure 2 and Figure 3). The patient reported that he did not follow up with oncology for adjuvant systemic therapy following the resection of the RPLPS. Suspicions were high for recurrent high-grade undifferentiated LPS with concern for metastases. After discussions with oncology and general surgery, the patient elected to undergo elective open excision resection of the abdominal mass. He was discharged after an effective bowel regimen was put in place to relieve his constipation and returned two and a half months later for the procedure. The surgical operation consisted of two en bloc resections involving the small bowel, resulting in two jejunojejunostomies. The pathological report characterized the surgical specimens as recurrent grade 3 DLPS, with multiple mesenteric tumor deposits. The ensuing hospital course was uneventful, and the patient was discharged to a short-term rehab facility to improve his strength and regain his independence. 

## Discussion

RPS are classified as heterogeneous tumors that arise in the retroperitoneal space of the abdomen [8]. They are organized into histological subtypes, including gastrointestinal stromal tumors (GISTs), leiomyosarcomas, and LPS, with the latter being the most common retroperitoneal tumor [7]. The benign histological tumor subtypes that may arise in the retroperitoneum include lipomas and hibernomas [7]. Differentiating between benign and malignant retroperitoneal tumors considers the location and histological features present [7]. Specifically, malignant tumors are four times more frequent in the retroperitoneum and display more cellular pleomorphism and nuclear atypia than their benign counterparts [7].

RLPS can be divided into two groups: well-differentiated and dedifferentiated. Dedifferentiated RLPS are associated with a higher risk of recurrence and metastasis [3,9]. They are particularly aggressive and can present with rapid growth and a high rate of local recurrence [6]. Leiomyosarcomas originate from smooth muscle and are recognized best for their tendency to metastasize to distant organs such as the lungs and liver [7,10]. This presents with symptoms specific to the respective system in which the tumor has metastasized, such as shortness of breath, cough, or right upper quadrant pain. 

The symptoms of RPS, however, can vary depending on the tumor's size, location, and interaction with neighboring structures. Symptoms in the early stages may be nonspecific; in many cases, patients can be asymptomatic until the tumor reaches a larger size [11]. Abdominal pain and back pain are common presenting symptoms due to RPS location in the retroperitoneum, which may compress adjacent organs, nerves, or tissues [9]. A palpable mass may be felt in the abdomen or flank as the tumor grows. Systemic symptoms such as weight loss and fatigue may present in patients with larger, more aggressive tumors or those with metastasis [12]. Gastrointestinal symptoms, including nausea and vomiting, can occur when the tumor involves the intestines, kidneys, or other structures in the retroperitoneum [13]. Hydronephrosis or renal dysfunction can result from the tumor pressing on the ureters, leading to obstruction of urine flow and causing kidney damage [13]. If hematuria is present, it indicates direct tumor involvement of the bladder and/or ureters [13].

Due to the insidious nature of this disease, diagnosis of RPS often occurs incidentally [8]. Imaging studies such as CT and MRI are essential for localizing the tumor and assessing its relationship to adjacent structures [5]. The lungs and the liver are the most common sites for metastasis, and early detection of metastatic disease is vital for improving patient prognosis [10]. Biopsies are the definitive method for diagnosing RPS and determining its histologic subtype [14]. A core needle biopsy or fine-needle aspiration is performed under imaging guidance, although there is a risk of tumor seeding along the biopsy track [3].

Given the nonspecific presentation of RPS, it is imperative to consider other differential diagnoses during the workup. These include germ cell tumors, lymphomas, and metastatic lesions from distant primary cancers such as the colon, pancreas, and lungs [8]. Benign diagnoses such as cysts, fibromas, lipomas, and retroperitoneal abscesses also share similar imaging characteristics with sarcomas and should be considered in the differential diagnosis [5]. A patient's clinical history, imaging studies, and biopsy results are crucial for distinguishing these entities [14].

The mainstay of treatment for RLPS is surgical resection [7]. Surgery aims to achieve negative margins while maximizing disease clearance and preserving surrounding organs and tissues [15]. Achieving negative margins is difficult given the proximity of these tumors to critical structures such as the kidneys, pancreas, and major blood vessels [5]. Neoadjuvant radiation therapy can be used to decrease the size of tumors before surgery [7]. The role of radiation remains limited as many sarcomas are resistant [10]. Chemotherapy response differs among RPS subtypes, such that leiomyosarcoma has been found to be chemosensitive, while other subtypes have controversial findings in response to chemotherapy treatment efficacy [16]. In contrast, targeted therapy, particularly tyrosine kinase inhibitors (TKIs) like imatinib, has shown promise in GISTs that metastasize to the retroperitoneum; they also play a role in the treatment of unresectable tumors [7]. Overall, the histological subtype of the tumor aids in determining the treatment modality.

RLPS have a high recurrence rate [17]. The risk of recurrence, ranging from 40% to 60% for previously treated LPS, must be considered during remission [2]. Even after achieving clear margins during surgical resection, the risk of recurrence remains high due to the infiltrative nature of RPS [18]. The retroperitoneum can be a challenging area to navigate, which contributes to the difficulty in achieving complete resection [18]. Factors such as tumor size, grade, and vascular invasion play an important role in predicting recurrence [19]. Furthermore, tumors with sizes greater than 5 cm, high-grade features, or concerning histopathology correlated significantly with metastatic recurrence [19]. Dedifferentiated RLPS have a higher tendency for recurrence and metastasis [6]. Incomplete surgical resection or failure to obtain negative margins is associated with remarkable increases in the likelihood of recurrence [7]. Recurrence typically occurs within the first 2-3 years after surgery [9]. These critical points highlight the necessity of frequent, long-term follow-up with imaging to detect any early signs of recurrence or metastasis [2,3].

In this case, the lack of regular follow-up care likely allowed for the recurrence to go undetected until the patient presented with symptoms [3]. Non-compliance with follow-up protocols remains a significant challenge in managing sarcomas, as routine imaging and clinical assessments are crucial for detecting early signs of recurrence [20].

## Conclusions

This case demonstrates the importance of early detection and long-term surveillance in the management of RPS. In addition, this case also accentuates the critical importance of consistent follow-up in sarcoma patients, particularly in the first few years after surgery when the risk of recurrence is highest. Regardless of aggressive initial treatment, patients with RPS must undergo long-term surveillance to detect recurrences or metastasis early, ideally before they become symptomatic. Despite the advances in surgical techniques and imaging, RPS remains a complex and challenging condition due to its high recurrence rates and the limited effectiveness of adjuvant therapies like chemotherapy. Treatments and therapies must be specific to each individual subtype, as characteristics and disease progression may vary. Nonetheless, surgical interventions followed up by routine observation and surveillance are paramount in providing long-term remission. The case highlights the importance of post-surgical care while depicting the consequences of neglecting such modalities.
